# Complications of traditional circumcision amongst young Xhosa males seen at St Lucy's Hospital, Tsolo, Eastern Cape, South Africa

**DOI:** 10.4102/phcfm.v5i1.488

**Published:** 2013-05-23

**Authors:** Ugochukwu Anike, Indiran Govender, John V. Ndimande, John Tumbo

**Affiliations:** 1 Family Physician, St Lucy's Hospital, South Africa; 2 Family Physician, University of Limpopo (Medunsa campus), South Africa

## Abstract

**Background:**

Traditional circumcision of males is common amongst many societies in sub-Saharan Africa. Circumcision amongst the Xhosa people of South Africa represents a rite of passage to manhood. Traditional male circumcision has an increased risk for complications that include sepsis, genital mutilation, gangrenous penis, excessive bleeding, dehydration, renal failure and death. The aim of this study was to describe the complications of traditional circumcisions amongst Xhosa men as seen at St. Lucy's Hospital in the Eastern Cape Province.

**Method:**

A cross-sectional descriptive quantitative study was conducted in 2008. Records of 105 males admitted to St. Lucy's Hospital with complications following traditional circumcision were reviewed. Data collected included age, education level, race, reasons for circumcision, complications, the period of circumcision, duration of hospital stay and the outcomes. Descriptive data analysis was performed using statistical software SPSS 17.0.

**Results:**

The ages ranged from 15–35 years with 68 (64.8%) between 15–19 years. 83 (79%) had a secondary level of education, 16 (15.2%) primary, 5 (4.8%) tertiary and 1% had no education. 60 (57%) were circumcised as initiation to manhood, 21 (20.0%) due to peer pressure, 20 (19.0%) for cultural reasons, and 1 (1.0%) was forced. The complications were sepsis (59 [56.2%]), genital mutilation (28 [26.7%]), dehydration (12 [11.4%]) and amputation of genitalia (6 [5.7%]).Fifty-nine (56.2%) patients were circumcised in winter. 79 (75.2%) were circumcised in the forest, and 25 (23.8%) in initiation centres. Fifty-eight (55.2%) were circumcised by traditionalists, and 47 (44.8%) by tribal elders (initiators). Hospital stays ranged from 8 to 28 days. 66% were healed and discharged, and 29 (27.6%) were referred to higher centres of care.

**Conclusion:**

Genital sepsis was the most common complication of traditional male circumcision. Complications were related to the circumciser, advanced age of the patient and place of circumcision. There is need for training of the traditional circumcisers on safe techniques and use of hygienic practices in order to reduce the complications identified in this study.

## Abstrait

**Les complications de la circoncision traditionnelle entre jeunes hommes Xhosa vu à l'Hôpital Sainte-Lucie, Tsolo, Eastern Cape, Afrique du Sud**

**Contexte:**

La circoncision traditionnelle des hommes est une pratique courante dans nombre de sociétés d'Afrique subsaharienne. Chez les Xhosa d'Afrique du Sud, la circoncision est un rite de passage vers l’âge adulte. La circoncision traditionnelle masculine est associée à un risque accru de complications, incluant les septicémies, les mutilations génitales, les abcès gangreneux du pénis, des saignements excessifs, la déshydratation, l'insuffisance rénale et la mort. L'objectif de cette étude était de décrire les complications découlant des circoncisions traditionnelles chez les hommes Xhosa à l'hôpital de St Lucy, dans la province du Cap oriental.

**Méthode:**

Une étude quantitative, descriptive et transversale a été réalisée en 2008. Les dossiers de 105 hommes admis à l'hôpital de St Lucy pour des complications associées à la circoncision traditionnelle ont été étudiés. Les données recueillies incluaient l’âge, le niveau d’éducation, la race, les raisons de la circoncision, les complications, la période à laquelle la circoncision avait été réalisée, la durée du séjour à l'hôpital et son issue. Une analyse descriptive des données a été réalisée à l'aide du logiciel de traitement statistique SPSS 17.0.

**Résultats:**

Les patients étaient âgés de 15 à 35 ans, 68 d'entre eux (64,8%) étant âgés de 15 à 19 ans. 83 (79%) avaient atteint l’école secondaire, 16 (15,2%) l’école primaire, 5 (4,8%) l'enseignement tertiaire et 1% n'avaient jamais été scolarisés. Soixante (57%) avaient été circoncis pour devenir des hommes, 21 (20%) en raison de la pression exercée par leurs pairs, 20 (19%) pour des raisons culturelles et un (1%) y avait été forcé. Les complications relevées étaient la septicémie (59 [56,2%]), les mutilations génitales (28 [26,7%]), la déshydratation (12 [11,4%]) et l'amputation des organes génitaux (6 [5,7%]). Cinquante-neuf (56,2%) patients avaient été circoncis l'hiver. Soixante-dix-neuf (75,2%) avaient été circoncis dans la forêt et 25 (23,8%) dans des centres d'initiation. Cinquante-huit (55,2%) avaient été circoncis par des traditionnalistes et 47 (44,8%) par des anciens tribaux (initiateurs). La durée des séjours à l'hôpital variait de 8 à 28 jours. Soixante-six pour cent étaient guéris et quittaient l'hôpital, et 29 (27,6%) étaient transférés vers des centres de soin de niveau supérieur.

**Conclusion:**

La septicémie des organes génitaux était la complication la plus courante associée à la circoncision traditionnelle masculine. Les complications étaient associées aux personnes procédant à la circoncision, à l’âge avancé du patient et au lieu de la circoncision. Il est nécessaire de former les circonciseurs traditionnels aux techniques sans dangers et à l'utilisation de pratiques hygiéniques afin de réduire la propension des complications identifiées dans cette étude.

## Introduction

### Background

Circumcision amongst Xhosa people represents a rite of passage that prepares the initiate for his transition to manhood. An unsterilised, unwashed blade may be used on a dozen or more initiates in a single session.^1^ Initiates are also significantly dehydrated during their 2-week period of seclusion in the belief that this reduces weeping of the wound, and after-care may be in the hands of a traditional attendant with no basic medical training.^1^ In some settings, initiates are rushed to the hospital, with the penile shaft covered in maize leaves or eucalyptus leaves – this may lead to infection.^2^

Traditionally, in Xhosa culture, a man could not marry until he had been initiated.^3^ Initiation conferred adult status, and an uncircumcised man could not inherit property.^3, 4, 5^

Circumcision is the resection of the foreskin at the level of the corona of the glans, whilst preserving enough frenulum to permit erection.^6^ Indications for circumcisions include: phimosis, paraphimosis, recurrent balanitis, recurrent urinary tract infections (UTIs) (in children), injury to the foreskin, and cosmetic reasons, including at the patients’ request. There are also social and cultural reasons for circumcisions.^6^

Traditional male circumcision has an increased risk for complications, such as sepsis, genital mutilation, gangrenous penis, excessive bleeding, death, septicaemia, gangrene, permanent disability from complete or partial amputation of the glans or shaft, the formation of a skin-bridge between the penile shaft and the glans, urinary retention, meatal ulcers, meatal stenosis, fistulae, loss of penile sensitivity, dehydration (which may result in renal failure), sexual dysfunction and oedema of the glans penis.^7^ Problems with traditional circumcision in the Eastern Cape led to legislation being promulgated in order to regulate the health standards – the Traditional Circumcision Act 6 of 2001.^8^

Circumcision by trained and experienced people is a safe procedure that effectively reduces cancer of the penis, urinary tract infection (UTI), sexually transmitted infections (STIs) and HIV amongst males.^9, 10, 11, 12, 13, 14^ Traditional circumcision is a cultural necessity which is at risk for fatal complications.

Thirteen of the 14 (93%) developing countries in North Africa and the Middle East have high male circumcision prevalence and being Muslim is strongly associated with male circumcision.^15^ In Africa, most circumcisions are performed by traditional and unqualified practitioners in informal settings.^16^

The Recent Joint United Nations Program on HIV/AIDS/World Health Organization (UNAIDS/WHO) policy proposed male circumcision for the prevention of HIV. About 15 million circumcisions are performed per year.^17^

Amongst the Babukusu, an ethnic group in Kenya, circumcision is part of the initiation rite for youths aged 8–20, and circumcision may be carried out traditionally or medically.^2^ Recently, young and inexperienced traditional circumcisers have been conducting the ritual. This has led to a high prevalence of deaths and mutilations as a consequence of botched surgery.^3^

#### Significance of the study

This study sought to establish the factors associated with the type and severity of complications from traditional circumcisions amongst Xhosa men as seen at St. Lucy's Hospital. St. Lucy's Hospital is situated in the Eastern Cape Province.

## Research methods and design

A cross-sectional descriptive quantitative study was conducted. The study population was all the male patients consulting at St. Lucy's Hospital following traditional circumcision from January 2006–December 2007. Data on complications of traditional circumcisions were collected from the selected (105) patient records from those of all male patients circumcised traditionally and admitted to hospital with complications within the period of the study. Demographic and medical information collected included age, occupation, race, reasons for circumcision, type of complications, the period of circumcision, duration of hospital stay and the outcomes. Anonymity of the records was maintained.

### Analyses

Descriptive data analysis was performed using statistical software SPSS 17.0. and presented in tables and figures.

**FIGURE 1:**
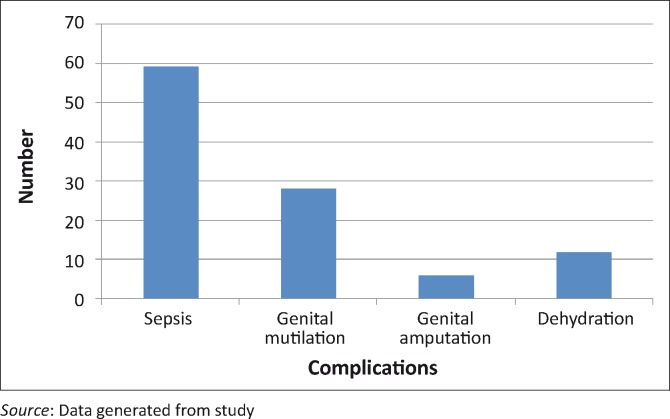
Complications of the circumcision.

**FIGURE 2:**
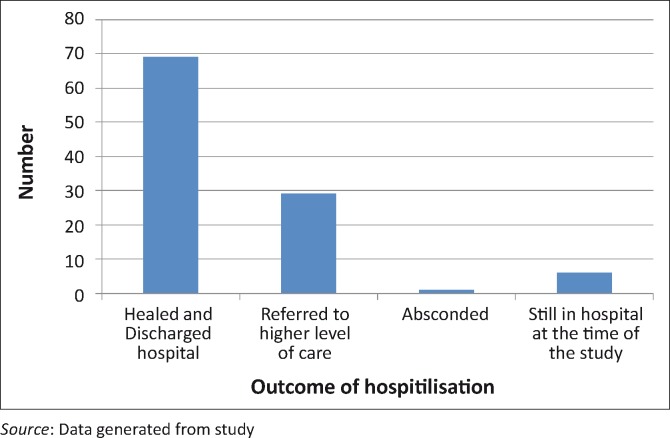
Outcome of hospitalisation due to complications of circumcision.

**TABLE 1:** Characteristics of participants.

Characteristics	Sub-characteristics	Total	
		
		*n*	%
Age (years)	15–19	68	64.5
	20–24	24	22.9
	25–29	9	8.6
	30–35	4	3.8
Highest Education level	No schooling	1	0.9
	Primary school	16	15.2
	Secondary School	83	79.0
	Tertiary education	5	4.5
Period of circumcision	Jan–Mar	2	1.8
	Apr–Jun	30	28.6
	Jul–Sep	59	56.2
	Oct–Dec	14	13.3
Place of circumcision	Forest	80	76.2
	Traditional initiation centre	25	23.8
Circumciser	Traditional circumciser	58	55.2
	Tribal elder	47	44.8
Reason for circumcision	Culture	23	21.9
	Peer pressure	21	20.0
	Forced	1	0.9
	Self-choice as rite to manhood	60	57.2
Number of days hospitalised	0–7	9	8.6
	8–14	53	50.5
	15–21	34	32.4
	> 22	9	8.6

*Source*: Data gathered during study *n*, Given as number.

## Results

There were a total number of 105 initiates with ages ranging from 15–35 years. The majority (68 [64.8%]) of the patients were between 15–19 years old, 24 (22.9%) were between 20–24 years old, 9 (8.6%) were between 25–29 years of age and 4 (3.8%) were aged between 30–35 years.

The majority of the patients had a secondary level of education (83 [79%]), 16(15.2%) had primary education only, 5 (4.8%) had tertiary education, and 1% had no education.

Of the 105 male patients, 60 (57%) were deduced to have been circumcised to become a man, 21 (20.0%) due to peer pressure, 20 (19.0%) due to culture, 1 (1.0%) was forced, and there was no information for 3% of the patients.

The complications were sepsis (59 [56.2%]), genital mutilation (28 [26.7%]), dehydration (12 [11.4%]) and amputation of genitalia (6 [5.7%]). No deaths were reported.

Fifty-nine (56.2%) patients were noted to have been circumcised in winter between July and September and 30(28.6%) between April and June. The highest number of patients were circumcised in the forest (80 [76.2%]), and 25 (23.8%) in an initiation centre. Fifty-eight (55.2%) were circumcised by a traditionalist, and 47 (44.8%) by tribal elders (initiators). The majority (53 [50.5%]) spend 8–14 days in hospital, whilst 34 (32.4%) spent 15–28 days. Approximately 66% were healed and discharged, but 29 (27.6%) were referred to higher centres of care.

## Ethical considerations

Permission to conduct the research was obtained from the management of St Lucy's Hospital. Approval for the study was granted by the University of Limpopo – Medunsa Research and Ethics Committee (MREC Certificate Number MREC/M/196/2008: PG).

## Discussion

Although there is increasing interest in investigating the complications with regard to traditional male circumcision, there is limited literature on this issue in sub-Saharan Africa. The inaccessibility of health authorities to the sites where traditional circumcisions are performed may explain the poor reporting and the patients’ culture will not generally accept that they present themselves to the health facilities with complications from such procedures.

In this study, sepsis (wound infection) was the most common circumcision complication that resulted in patients seeking medical intervention. Fifty-nine (56.2%) of the total 105 patients had wound sepsis, followed by genital mutilation which occurred in 28 (26.7%) patients. Twelve (11.4%) suffered from dehydration and 6 (5.7%) had amputation of the glans. These findings were similar to those of a previous study in the Eastern Cape.^2^ The following major adverse events were found: 20.8% had mild delayed wound healing, 16.2% had a mild wound infection, 10.5% had mild pain and 10.4% had insufficient skin removed.2 Wound infection (sepsis) was again the most frequent complication.

In Kenya, 21% of the patients undergoing circumcision had delayed healing and 10% had sepsis.^16^ Of 479 medically circumcised male adults in Kenya, only 3.5% were associated with adverse events.^18^ The most common adverse events were wound infections (1.3%).^18^ This is also in keeping with our study which recorded sepsis as the most common complication. In our study those with serious infections stayed longer in the hospital and those with extensive mutilation were referred for further care to tertiary hospitals.

Circumcision in Nigeria and Kenya was carried out by untrained traditional circumcisers in 80% of cases. One patient died from septicaemia, 2 lost their penises to gangrene, and 5 had permanent disability from either complete or partial amputation of the glans or shaft.7 Another study in Nigeria found that the most common complications were haemorrhage and infection.^19^

In Turkey, with 407 men surveyed, complications were seen in 73% of cases, with the most common complications being wound infection, subcutaneous cysts, bleeding, and haematoma. Five men developed a urinary infection requiring hospitalisation and intravenous antibiotics.^20^ In our study, the majority of patients (64.8%) were between 15 and 19 years of age and wound infections were also the most common complication.

There is wide variation in the reported frequencies of adverse events following circumcision. This is likely to be due to several factors directly associated with complications such as age at circumcision, training and expertise of the provider, the sterility of the conditions under which the procedure is undertaken, and the indication (medical or cultural) for circumcision.

Bleeding was not seen as the major complication in this study because the initiates usually stayed two weeks or more in the forests and/or initiation centres, before they were released. Sepsis may be attributable to the unsterile environment in which the circumcisions were performed, or poor wound care.

As is the practice amongst the Xhosa in South Africa (SA), an unsterilised, unwashed blade may be used on many initiates, many are dehydrated during their seclusion in the belief that this reduces weeping of the wound, and after-care may be in the hands of a traditional attendant.^2^

Dehydration was a result of reduced intake of fluids and because the initiates were kept at outdoor camps or mountains, sometimes for weeks, with minimal or inadequate shelter, especially during summer, which can lead to increased sweating leading to dehydration and possible renal failure. Many of the men admitted had to be rehydrated intravenously.

Amputation of the glans could have occurred because the glans was not well shielded from the sharp edge of the instrument used, and incorrect instruments may also have been used. In a study by Karl Peltzer et al., they noted that more than half (53%) of the traditional circumcisers had used the culturally more acceptable but medically less safe assegai (spear) for the circumcision, which may have contributed to some of the circumcision complications.^2^

Circumcision is usually carried out amongst the Xhosa using a razor blade or penknife without anaesthesia. The wound is covered with eucalyptus leaves or sometimes maize leaves and left in place for four weeks whilst the men are in seclusion.^2^

The tight dressing to provide haemostasis often causes ischaemia, which leads to gangrene and loss of the glans. It was noted that some of the penile wounds were wrapped in a piece of cloth and others with leaves.

Death was not recorded in our study probably because those that were in a critical condition were referred to a higher level of care. Septicaemia is the most common cause of death from traditional circumcisions.^21^

There is considerable variation in the age at which circumcision takes place, which may affect the outcome. Neonatal circumcision is common in West Africa, but is uncommon in East and Southern Africa where the median age at circumcision varies from boyhood to the late teens.^2^

In our study most men (64.8%) were circumcised between 15–19 years of age. In a similar study in SA, 84% of the respondents were adolescents with a mean age of 20 years.^22^ In another study in SA, the median age of circumcision for Black males was 18 years as compared with 10 years for Coloured males, 2 years for White males and 1 year for Indian males.^23^

In South Korea, of 1306 men circumcised, 7.8% were circumcised at age <10 years and 15.0% at >15 years. In the group 10–15 years, 55.2% were circumcised. About 1.0% of men were circumcised within 1 year of birth. In Pakistan, male babies are circumcised a few days before discharge, whereas those born outside hospital are circumcised between the ages of 3 and 7.^24^

Cultural beliefs and tradition play a strong role in the reasons for circumcision. The majority of males (60 [57%]) went for circumcisions to become a man in accordance with their culture. In another South African study the majority went for circumcisions because their friends refer to them as ‘boys’.^22^ It is common for uncircumcised men to be labelled by other men during discussions pertaining to manhood.^23^ Some elderly men have to undergo circumcision later in life to finally gain acceptance from other males.^23^

Most circumcisions (56.2%) were performed during the winter months. This is in keeping with other studies noting that traditional male circumcisions in Africa occur mainly during the winter season.^3^

As with other studies, the circumcisers have a lot to do with the outcome of traditional circumcision.^2, 3^ In this study it was shown that complications were more prevalent when the circumciser was a traditionalist. The total rate of complications was 54.3% when a traditionalist was involved, compared to 28.6% when a tribal elder was involved. These findings did not differ from previous studies.^24^

It was shown that the number of admission days is dependent on the severity and the type of complications involved. Those that were discharged within 3–7 days had minor sepsis. One of the participants with amputation of the glans stayed 3–7 days before being referred to a higher level of care for possible reconstruction.

## Limitations of the study

This study relied on information from the patient records, some which were incomplete. Due to the small sample size, it was not possible to demonstrate any association between the variables and the complications.

## Conclusion

From our study, genital sepsis was the most common complication of traditional male circumcision. Complications were related to the circumciser, advanced age of the patient and place of circumcision. There is need for training of the traditional circumcisers on safe techniques and use of hygienic practices in order to reduce the complications identified in this study. Health authorities need to collaborate with the tribal authorities so as to mitigate the complications of this culturally-driven circumcision.
